# Risk and Protective Factors in Autism Spectrum Disorders: A Case Control Study in the Lebanese Population

**DOI:** 10.3390/ijerph17176323

**Published:** 2020-08-31

**Authors:** Perla Gerges, Tania Bitar, Mirna Hawat, Abbas Alameddine, Michel Soufia, Christian R. Andres, Walid Hleihel

**Affiliations:** 1 Faculty of Arts and Sciences, Holy Spirit University of Kaslik (USEK), Jounieh 446, Lebanon; mirna.c.hawat@net.usek.edu.lb (M.H.); walidhleihel@usek.edu.lb (W.H.); 2 UMR Inserm 1253 Ibrain, Université de Tours, 37032 Tours, France; christian.andres@univ-tours.fr; 3 Department of Psychology, University of Balamand, 100 Tripoli, Lebanon; abbas.alameddine@gmail.com; 4 North Autism Center (NAC), Zgharta 1304, Lebanon; 5 School of Medicine and Medical Sciences, Holy Spirit University of Kaslik (USEK), Jounieh 446, Lebanon; michel_soufia@hotmail.com

**Keywords:** Autism spectrum disorders, protective factors, multivitamins, cereal, iron, risk factors, attention deficit disorder, stress

## Abstract

Autism spectrum disorders (ASD) are among the most common childhood neurodevelopmental disorders. Identification of risk and protective factors are necessary to improve the guidance of prevention and intervention strategies. Our study aims to determine the potential risk and protective factors in ASD in the Lebanese population. Our case-control study included 100 ASD patients and 100 healthy matched controls recruited from all the Lebanese districts. The data collected from the questionnaires was analyzed using SPSS 23.0. Independent Student T-test and Chi-Square test were carried out for the bivariate analysis of the data. In addition, the variables revealing a *p*-value < 0.05 were used for the multivariate logistic regression analysis. Multivitamins intake, especially omega 3 and vitamin B (Odds Ratio (OR) = 0.257; 95% Confidence Interval (CI) [0.115–0.579]), rich cereal diet (OR = 0.212; 95% CI [0.089–0.510]), and supplementation in iron during pregnancy (OR = 0.229; 95% CI [0.083–0.627]) were identified as protective factors against ASD. On the other hand, stress during pregnancy (OR = 6.339; 95% CI [2.845–14.125]), the presence of ASD patients in the family (OR = 7.878; 95% CI [1.877–33.065]) and the presence of attention deficit hyperactivity disorder (ADHD) patients in the family (OR = 6.981; 95% CI [1.362–35.789]) were associated with ASD. This study shed light on risk and protective factors associated with ASD in the Lebanese population. Further rigorous research, taking into consideration these factors, is needed to assist in early detection, prevention and subsequent intervention targeting ASD and its associated comorbidities, given that our study is not experimental and does not prove causality.

## 1. Introduction

Autism spectrum disorders (ASD) are defined as complex, neurodevelopmental conditions characterized by impairment in reciprocal social communication, restrictive and repetitive behaviors [1], reflecting the interaction between genetics and environmental risk factors [2,3]. Worldwide, the prevalence of ASD is estimated to affect 1/160 according to the World Health Organization (WHO) [2]. However, the prevalence is estimated to be 1/66 children in Lebanon [4].

In fact, genetic abnormalities in more than 1000 genes which are important for synaptic function, ubiquitination and chromatin remodeling have been identified in ASD [5]. Furthermore, the diverse observed phenotypes in ASD patients suggest that environmental factors play a role in the manifestation of the disorder in genetically susceptible subjects. To date, several environmental factors have been mentioned for ASD [6]. Exposure of the mother and the child to harmful environmental agents during central nervous system development can change the expression of some genes that are essential to embryonic development, leading to an increased risk of developmental disorders which are frequently associated with ASD [7].

Such findings, emphasizing the contribution of the environment in the etiology of ASD, strengthen the importance of identifying risk and protective factors in order to minimize the deleterious interaction between environmental and genetic factors. Several studies have proven that some parental, prenatal and postnatal factors increase the risk of ASD [8,9]. For example, a significantly higher incidence of ASD has been observed in children having mothers without folic acid supplementation during pregnancy compared to whom had their mothers supplemented [10]. As for iron, it has been reported that its deficiency, especially during breastfeeding, could lead to an increased ASD risk [11]. To date, there is no curative treatment for ASD. However, associated comorbidities could be reduced by prenatal measures and early behavioral interventions [12,13].

Considering that the environmental factors of ASD are not fully elucidated, our study which was conducted in the Lebanese population focused on identifying protective and risk factors that can help in designing future studies aiming for ASD prevention or risk attenuation.

## 2. Materials and Methods

### 2.1. Participants

This case-control study included 100 ASD patients and 100 matched controls aged between 1 and 31 years old recruited from specialized institutions covering all the Lebanese territories between 2015 and 2020. With a confidence level of 95% and a power of 80%, we wanted to be able to detect a difference of at least 15% in the distribution of the factors between the two groups; therefore, we needed at least 99 persons per group. ASD patients were diagnosed using the fifth edition of the Diagnostic and Statistical Manual of Mental Disorders (DSM V).

### 2.2. Recruitment and Data Collection

Our research team contacted specialized organizations working with ASD patients to explain the objectives of the study. Following the call, a letter demonstrating the objectives of our study was sent to the families, followed by a meeting during which two members of the team answered the questions of the parents. The parents who accepted the invitation to participate in the study signed an informed consent and filled a questionnaire by either the mother or the father, or sometimes both under the supervision of a member from the team with the highest standards of confidentiality (see Supplementary File S1).

Simultaneously, the same procedure used in the recruitment of ASD patients was used to recruit the control group. The research team contacted regular schools and explained the objectives and stages of the study. After signing the informed consent, the surveys were filled out by either one or both of the parents in the presence of a member of our team (see Supplementary File S2). The control group matched the ASD group for age. According to the answers of the survey, control subjects did not have any neurodevelopmental disorders and they were socially active.

### 2.3. Variables and Trial Design

The retrospective survey, covering demographic, prenatal and postnatal factors, was prepared by our team according to the findings of available studies on ASD. It was conducted among the spoken language, which is Arabic.

Demographic factors such as the participant’s age and parental age at conception were investigated. In addition, the parental and sibling’s medical history was considered for: ASD, attention deficit hyperactivity disorder (ADHD), dyspraxia, and autoimmune diseases.

As for the prenatal factors, maternal stress, multivitamins intake, iron supplementation, rich cereal diet, coffee, tea and alcohol consumption, and passive exposure to tobacco at home during pregnancy were all checked. The binary “Yes/No” questions concerning these factors were directed at the conception period.

The associated disorders in ASD patients were also investigated for: digestive disorders, allergic reaction to some foods, mental retardation, and epilepsy.

This case control study has complied with the ethical standards and guidelines declared by the Declaration of Helsinki in 1964 and its later amendments and approved by the Holy Spirit University Ethical Committee after reviewal (delivered on 13 August 2014 and renewed on 9 January 2019).

### 2.4. Statistical Analysis

SPSS software version 23.0 (IBM SPSS Inc., Chicago, IL, USA) was used for the analysis. Student independent *t*-test was carried out to compare means in two groups. Chi-Square test was used to compare the distributions in two groups. A *p*-value < 0.05 was considered significant. Moreover, the multivariate logistic regression analysis was achieved using variables with *p* < 0.05 in the bivariate analysis and the absence/presence of ASD was used as the dependent variable. Odds ratios (OR) are also reported within 95% confidence intervals (CI) to determine whether the variable is a risk or protective factor.

## 3. Results

Our study showed a predominance of 84% male ASD patients compared to 16% female ASD patients. Furthermore, the independent student *t*-test was performed for the participant age and parental age at conception which showed a non-significant difference in all ages for both groups as shown in Table 1.

It is widely known that ASD have many associated disorders [14]. Moreover, our results revealed that several disorders such as digestive disorders, intellectual disability and epilepsy are present in the ASD patients of our study. Intellectual disability is shown to be the most common condition in ASD patients (44%), as shown in Table 2.

### 3.1. Bivariate Analysis

Our results, presented in Table 3, showed a statistical significance between ASD familial history and ASD onset. A slightly higher percentage of ASD patients compared to controls had an ASD brother/sister (8% versus 1%), ASD patients in their parent’s families (12% versus 4%), ADHD patients in the family (14% versus 2%) and dyspraxic children in the family (8% versus 0%). As for the prenatal factors, our study revealed a significant *p*-value for stress during pregnancy, passive exposure to tobacco at home, multivitamins intake and cereal diet.

### 3.2. Multivariate Analysis

The variables included in the multivariate analysis were the ones having a *p*-value < 0.05 in the bivariate analysis. The presence/absence of ASD was taken as the dependent variable, whereas the independent variables are shown in Table 4. Our results have shown that a rich cereal diet (OR = 0.212), multivitamins intake (especially omega 3 and vitamin B (OR = 0.257)) and iron supplementation during pregnancy (OR = 0.229) were identified as protective factors for ASD occurrence. Whereas reported mother stress during pregnancy (OR = 6.339), the presence of ASD patients in the family (OR = 7.878) and the presence of ADHD patients in the family (OR = 6.981) are risk factors for ASD. Our analysis showed no association between the history of autoimmune diseases in the family and maternal passive tobacco exposure at home during pregnancy and ASD.

## 4. Discussion

This case control study was conducted to determine the prenatal, postnatal, and familial factors related to ASD in Lebanese individuals.

Our study uncovered significant protective factors that could easily be promoted with the objective to reduce the risk of ASD. In our cohort, mothers who reported taking multivitamins, including omega 3 and vitamin B, were less likely to have children with ASD. In parallel with previous studies, prenatal vitamins supplementation was associated with decreased risk of ASD occurrence [15,16]. Pregnancy multivitamins are a combination of many vitamins including omega 3, omega 6, folic acid, iron, and vitamin B. Studies have revealed that vitamin supplementation rich in vitamin B prevents neural tube defects and improves the DNA methylation process [17]. Moreover, maternal deficiency in some vitamins during pregnancy is associated with aberrations in the neurodevelopment of the fetus. For instance, prenatal vitamin D deficiency causes the disruption of structural brain connectivity [15] and increases the risk of genetic mutations [10], which may lead to ASD and intellectual disability in offspring.

In addition, our findings showed that rich cereal diet during pregnancy constitutes a protective factor for ASD. Cereals are an essential source of a variety of nutrients, such as iron, folic acid and vitamins of the B group [18]. In agreement with the results of our study, a study examining maternal iron intake in relation to ASD risk in California showed that low maternal iron intake in addition to other factors, such as advanced maternal age, was associated with a 5-fold increase in ASD risk [11].

One more protective factor showed in our study is iron supplementation during pregnancy. A study conducted on the Lebanese population has shown that prenatal anemia is a risk factor associated with ASD [19]. Moreover, a recent study has shown that maternal anemia during pregnancy is highly associated with neurodevelopmental disorders, including ASD, attention deficit/hyperactivity disorder (ADHD) and intellectual disability (ID) in offspring [20]. Furthermore, as mentioned previously, Schmidt et al. reported that low iron intake was associated with ASD [11]. Indeed, prenatal anemia is associated with several complications affecting the maternal and fetal well-being. Affected mothers experience fainting and sleep difficulties, and they have an increased risk of developing infections. As for the fetus, iron deficiency has adverse outcomes, including growth delay and prematurity. Therefore, iron is crucial for neural metabolism and functioning [21].

In addition to the cited protective factors, our study uncovered three risk factors. The results of our study were in line with previous reports showing that maternal stress during pregnancy is a risk factor highly associated with ASD [22,23]. Indeed, many studies have shown the effects of prenatal exposure to maternal stress on the behavior and mental health of the offspring. These effects include abnormalities in neurodevelopment, neurocognitive function, and cerebral processing which lead to changes in both the hypothalamo–pituitary–adrenal axis (HPA) and the autonomic nervous system [24]. Corticotropin-releasing hormone (CRH) secretion from the hypothalamus is a result of stress and correlated with the level of stress during pregnancy. Maternal CRH can easily cross the placenta, and the placenta itself can produce considerable amounts of CRH due to external or intrauterine stress. CRH has an immunomodulatory role; it stimulates the activation of mast cells to release pro-inflammatory cytokines, such as interleukin 6 (IL-6), that increase blood–brain barrier (BBB)’s permeability. These events lead to the action of auto-antibodies against brain peptides, which cause brain inflammation, thus contributing to ASD pathogenesis [25].

Our findings also indicated that familial ASD history is more common among ASD group compared to controls. Our multivariate analysis revealed that having ASD and ADHD patients in the family are risk factors related to ASD, which support the role of genetics in ASD. In fact, a study conducted on a large cohort of Swedish children showed that the heritability of the disorder is approximately 50% and the risk of ASD increases 10-fold if a sibling has the disease and 2-fold if a cousin is diagnosed with ASD [26]. However, this result was concordant with the finding of two Lebanese studies showing that familial history of mental illness and ASD are significantly associated with ASD onset in children [23,27].

Despite the limited sample size of our case-control study, we were able to suggest not only risk factors for ASD but also protective factors. However, our study was of the retrospective type based on the information given from the parents, which leads to the possibility of memory bias and low conclusiveness. In addition, it was difficult to have a proportional geographic sampling since our recruitment was done in centers and schools that do not follow a strict and specific geographic distribution. The two studied groups differ widely in gender distribution, which constitutes a major limitation to our study; hence, a high proportion of patients with intellectual disability comorbidity were present in the ASD group. Another limitation was the difficulty to verify the diagnosis criterion because it was not done by the research team. Thus, taking into consideration the fact that our results come from an observational/correlational non-experimental work, causality is not inferred, and readers are warned to avoid formulating the study results into quick intervention guidelines. The aforementioned limitations should be taken into consideration for complementary studies using a larger population with more precise exposure assessments and potential cofounders.

## 5. Conclusions

ASD is one of the most prevalent childhood neurodevelopmental disorders around the world. Currently, all the Lebanese epidemiological studies conducted on ASD patients have a limited sample size compared with other countries. Subsequently, ASD epidemiological investigation on the Lebanese population should be improved in order to achieve more robust conclusions.

In short, our study suggested that maternal vitamin intake (especially vitamin B group and omega n-3 fatty acids), a cereal-rich diet, and iron supplementation during pregnancy are protective factors in ASD. On the other hand, several risk factors highly associated with ASD were reported, including maternal stress during pregnancy, the presence of ASD and ADHD patients in the family. Identifying these risk and protective factors associated with ASD will not only help us understand its prevalence but may also constitute important preventive measures.

Future studies with large and heterogeneous sample sizes should be done to identify the time of the exposure to environmental factors and its relation with critical developmental phases.

## Figures and Tables

**Table 1:** Demographic characteristics of control and Autism Spectrum Disorders (ASD) group.

Variable	Control Group	ASD Group	*p*-Value
Gender			0.000 *
Male	55 (55%)	84 (84%)	
Female	45 (45%)	16 (16%)	
Age			0.731
Mean ± standard deviation	10.40 ± 5.21	10.14 ± 5.51	
Mother’s age at conception			0.551
Mean ± standard deviation	28.52 ± 5.75	29.02 ± 6.19	
Father’s age at mother’s conception			0.716
Mean ± standard deviation	35.77 ± 8.36	36.17 ± 7.13	

* Significant *p*-value < 0.05.

**Table 2:** ASD comorbid disorders.

Variable	Control Group	ASD Group
Digestive disorders		
Yes	20 (20%)	33 (33%)
No	80 (80%)	67 (67%)
Allergic reaction to food		
Yes	3 (3%)	5 (5%)
No	97 (97%)	95 (95%)
Intellectual disability		
Yes	0 (0%)	44 (44%)
No	100 (100%)	56 (56%)
Epilepsy		
Yes	0 (0%)	11 (11%)
No	100 (100%)	89 (89%)

**Table 3:** Factors by bivariate analysis for ASD.

Variable	Control Group	ASD Group	*p*-Value
Maternal stress during pregnancy			0.000 *
Yes	18 (18%)	47 (47%)	
No	82 (82%)	53 (53%)	
Multivitamins intake during pregnancy			0.005 *
Yes	85 (85%)	68 (68%)	
No	15 (15%)	32 (32%)	
Iron supplementation during pregnancy			0.027 *
Yes	24 (24%)	12 (12%)	
No	76 (76%)	88 (88%)	
Coffee consumption during pregnancy			0.086
Yes	36 (36%)	48 (48%)	
No	64 (64%)	52 (52%)	
Tea consumption during pregnancy			0.079
Yes	31 (31%)	43 (43%)	
No	69 (69%)	57 (57%)	
Alcohol consumption during pregnancy			0.081
Yes	0 (0%)	3 (3%)	
No	100 (100%)	97 (97%)	
Cereal diet during pregnancy			0.001 *
Yes	88 (88%)	68 (68%)	
No	12 (12%)	32 (32%)	
Passive exposure to tobacco at home during pregnancy			0.008 *
Yes	30 (30%)	47 (47%)	
No	70 (70%)	53 (53%)	
ASD brother/sister			0.017 *
Yes	1 (1%)	8 (8%)	
No	99 (99%)	92 (92%)	
ASD children in the family			0.037 *
Yes	4 (4%)	12 (12%)	
No	96 (96%)	88 (88%)	
ADHD children in the family			0.002 *
Yes	2 (2%)	14 (14%)	
No	98 (98%)	86 (86%)	
Dyspraxic children in the family			0.004 *
Yes	0 (0%)	8 (8%)	
No	100 (100%)	92 (92%)	

* Significant *p*-value < 0.05.

**Table 4:** Logistic regression analysis taking the presence/absence of ASD as dependent variable.

Variable	*p*-Value	Odds Ratio (95% Confidence Interval)
Risk Factors		
Maternal stress during pregnancy	0.000 *	6.339 (2.845–14.125)
Presence of ASD patients in the family	0.005 *	7.878 (1.877–33.065)
History of autoimmune diseases in the family	0.206	4.534 (0.435–47.249)
Presence of ADHD patients in the family	0.020 *	6.981 (1.362–35.789)
Maternal passive tobacco exposure at home during pregnancy	0.394	1.174 (0.812–1.699)
Protective Factors		
Maternal rich cereal diet during pregnancy	0.000 *	0.212 (0.089–0.510)
Iron supplementation during pregnancy	0.004 *	0.229 (0.083–0.627)
Maternal multivitamins intake during pregnancy	0.001 *	0.257 (0.115–0.579)

Variable(s) entered in the model: Maternal stress during pregnancy, presence of ASD patients in the family, history of autoimmune diseases in the family, presence of ADHD patients in the family, maternal passive tobacco exposure at home during pregnancy, maternal rich cereal diet during pregnancy, iron supplementation during pregnancy and maternal vitamin intake during pregnancy. * Significant *p*-value < 0.05.

## Supplementary Materials

The following are available online at https://www.mdpi.com/1660-4601/17/17/6323/s1, File S1: Survey for ASD group, File S2: Survey for control group.

## References

[1] American Psychiatric Association (2013). Diagnostic and Statistical Manual of Mental Disorders.

[2] Eissa N., Al-Houqani M., Sadeq A., Ojha S.K., Sasse A., Sadek B. (2018). Current enlightenment about etiology and pharmacological treatment of autism spectrum disorder. Front. Neurosci..

[3] Siu M.T., Weksberg R. (2017). Epigenetics of autism spectrum disorder. Adv. Exp. Med. Biol..

[4] Chaaya M., Saab D., Maalouf F.T., Boustany R. (2016). Prevalence of autism spectrum disorder in nurseries in lebanon: A cross sectional study. J. Autism. Dev. Disord..

[5] SFARI | Simons Foundation Autism Research Initiative. https://www.sfari.org/.

[6] Forsberg S.L., Ilieva M., Maria Michel T. (2018). Epigenetics and cerebral organoids: Promising directions in autism spectrum disorders. Transl. Psychiatry.

[7] Karimi P., Kamali E., Mousavi S.M., Karahmadi M. (2017). Environmental factors influencing the risk of autism. J. Res. Med. Sci..

[8] Hornig M., Bresnahan M.A., Che X., Schultz A.F., Ukaigwe J.E., Eddy M.L., Hirtz D., Gunnes N., Lie K.K., Magnus P. (2018). Prenatal fever and autism risk. Mol. Psychiatry.

[9] Wang C., Geng H., Liu W., Zhang G. (2017). Prenatal, perinatal, and postnatal factors associated with autism: A meta-analysis. Medicine (Baltimore).

[10] Emberti Gialloreti L., Mazzone L., Benvenuto A., Fasano A., Garcia Alcon A., Kraneveld A., Moavero R., Raz R., Riccio M.P., Siracusano M. (2019). Risk and protective environmental factors associated with autism spectrum disorder: Evidence-based principles and recommendations. J. Clin. Med..

[11] Schmidt R.J., Tancredi D.J., Krakowiak P., Hansen R.L., Ozonoff S. (2014). Maternal intake of supplemental iron and risk of autism spectrum disorder. Am. J. Epidemiol..

[12] Reichow B., Hume K., Barton E.E., Boyd B.A. (2018). Early intensive behavioral intervention (EIBI) for young children with autism spectrum disorders (ASD). Cochrane Database Syst. Rev..

[13] Zachor D.A., Curatolo P. (2014). Recommendations for early diagnosis and intervention in autism spectrum disorders: An italian-israeli consensus conference. Eur. J. Paediatr. Neurol..

[14] Tye C., Runicles A.K., Whitehouse A.J.O., Alvares G.A. (2018). Characterizing the interplay between autism spectrum disorder and comorbid medical conditions: An integrative review. Front. Psychiatry.

[15] Levine S.Z., Kodesh A., Viktorin A., Smith L., Uher R., Reichenberg A., Sandin S. (2018). Association of maternal use of folic acid and multivitamin supplements in the periods before and during pregnancy with the risk of autism spectrum disorder in offspring. JAMA Psychiatry.

[16] Raghavan R., Riley A.W., Volk H., Caruso D., Hironaka L., Sices L., Hong X., Wang G., Ji Y., Brucato M. (2018). Maternal multivitamin intake, plasma folate and vitamin B12 levels and autism spectrum disorder risk in offspring. Paediatr. Perinat. Epidemiol..

[17] Imbard A., Benoist J.F., Blom H.J. (2013). Neural tube defects, folic acid and methylation. Int. J. Environ. Res. Public Health.

[18] Laskowski W., Górska-Warsewicz H., Rejman K., Czeczotko M., Zwolińska J. (2019). How important are cereals and cereal products in the average polish diet?. Nutrients.

[19] Guisso D., Saadeh F., Saab D., El Deek J., Chamseddine S., El Hassan H., Majari G., Boustany R. (2018). Association of autism with maternal infections, perinatal and other risk factors: A case-control study. J. Autism Dev. Disord..

[20] Wiegersma A.M., Dalman C., Lee B.K., Karlsson H., Gardner R.M. (2019). Association of prenatal maternal anemia with neurodevelopmental disorders. JAMA Psychiatry.

[21] Abu-Ouf N., Jan M. (2015). The impact of maternal iron deficiency and iron deficiency anemia on child’s health. Saudi Med. J..

[22] Beversdorf D.Q., Stevens H.E., Jones K.L. (2018). Prenatal stress, maternal immune dysregulation, and their association with autism spectrum disorders. Curr. Psychiatry Rep..

[23] Bitar T., Gerges P., Kassab M., Hallit S., Mattar H., Soufia M., Andres C.R., Hleihel W. (2020). Factors associated with autism spectrum disorder: A case-control study in the lebanese population. Epidemiol. Biostat. Public Health.

[24] Van den Bergh B.R.H., Van den Heuvel M.I., Lahti M., Braeken M., De Rooij S.R., Entringer S., Hoyer D., Roseboom T., Räikkönen K., King S. (2017). Prenatal developmental origins of behavior and mental health: The influence of maternal stress in pregnancy. Neurosci. Biobehav. Rev..

[25] Angelidou A., Asadi S., Alysandratos K., Karagkouni A., Kourembanas S., Theoharides T.C. (2012). Perinatal stress, brain inflammation and risk of autism-review and proposal. BMC Pediatr..

[26] Sandin S., Lichtenstein P., Kuja-Halkola R., Larsson H., Hultman C.M., Reichenberg A. (2014). The familial risk of autism. JAMA.

[27] Saab D., Chaaya M., Boustany R.M. (2018). National prevalence and correlates of autism: A lebanese cross-sectional study. Autism Open Access.

