# Supplementary material for: Risk and Protective Factors in Autism Spectrum Disorders: A Case Control Study in the Lebanese Population
Source: Int J Environ Res Public Health. 2020 Aug 31;17(17):6323. doi: 10.3390/ijerph17176323 (PMC7504462; doi:10.3390/ijerph17176323)
Supplement: Supplementary file 1 [file ijerph-17-06323-s001.zip › Supplementary File 1.pdf]

Informed consent

Holy Spirit University of Kaslik
Faculty of Arts and Sciences

I, the undersigned ...., parent guardian of ...., acknowledge that I am aware and certain that my son/daughter will participate in the Autism Spectrum Disorders (ASD) study carried out by the Holy Spirit University of Kaslik in Lebanon, and divided into three stages.

In the first stage, a detailed survey should be filled out about the participant medical condition and lifestyle. The information provided will remain confidential with the right to agreeing or refusing to answer questions.

In the second stage, which can be done in conjunction with the first stage, urine and fecal samples will be taken to perform the metabolic and microbiological studies.

As for the third and last stage, which can be done in conjunction with the first and second stage, blood samples will be taken from the person with ASD and his parents to conduct a detailed genetic study.

I agree for the participation of my son/daughter in the following stages:

- ☐ Fill in the form
- ☐ Providing urine samples
- ☐ Providing fecal samples
- ☐ Providing blood samples

I have the right to withdraw from the study whenever I want without giving an explanation and without any change in my relationship with the doctor, knowing that the samples, especially the genetic ones, will only be used for this purpose and I reserve the right to request their destruction after use.

Signature:

Signature of principal investigator of the study:

Date:

Autism in Lebanon

Family name<br>--------------------------------------	Name of the center/school/NGO<br>--------------------------------
Phone number<br>--------------------------------------	Date<br>-------------------------------

Survey

1- **Mother's age at conception :** ______________________________

2- **Father's age :** _____________________________________

3- **Participant's age :** ________________________________

4- **Participant's gender** :

Male ______ female ______

Questions related to the parents:

5- **Is there any ASD case in the family?**

Yes :_____ No: ____

If yes, specify the degree of consanguinity: ____________

6- **Is there any immunodeficiency disease case in the family?**

Yes: _____ No: ____

Questions related to the mother during pregnancy:

7- **Was the mother subjected to stress during pregnancy (death of a relative, war, family or marital problems, partner's death, loss of work ....)**

Yes :_____ No: ____

If yes, specify __________________

8- **Was the mother consuming caffeine during pregnancy?**

Yes: _____ No: ____

If yes, determine the quantity by day/week/month: __________________

9- **Was the mother consuming tea during pregnancy?**

Yes :_____ No: ____

If yes, determine the quantity by day/week/month: _________________

10- **Was the mother drinking alcohol during pregnancy?**

Yes: _____ No: ____

If yes, determine the quantity by day/week/month: __________________

11- **Did the mother take multivitamins during pregnancy?**

Yes :_____ No: ____

If yes, specify: __________________

12- **Was the mother supplemented with iron during pregnancy?**

Yes: _____ No: ____

13- **Was the maternal diet during pregnancy rich in cereals?**

Yes: _____ No: ____

14- **Was the mother exposed indirectly to tobacco at home?**

Yes: _____ No: ____

If yes, specify the duration of the exposure:

- between 1 and 2 hours____________

- between 2 and 4 hours______________

- more than 4 hours___________________

Questions related to the participant:

15- **Does any of the participant's sister/brother suffer from ASD?**

Yes: _____ No: ____

If yes, specify: ___________

16- **Do any of the children in the family suffer from:**

- Attention deficit hyperactivity disorder (ADHD):

Yes: _____ No: ____

- Dyspraxia:

Yes: _____ No: ____

17- **Does the participant suffer from digestive disorders such as constipation, diarrhea, stomach ache, nausea and gastric reflux?**

Yes: _____ No: ____

If yes, specify: ___________

18- **Does the participant have allergic reaction on food?**

Yes: _____ No: ____

If yes, specify: ___________
